# Simultaneous PET/MRI with ^13^C magnetic resonance spectroscopic imaging (hyperPET): phantom-based evaluation of PET quantification

**DOI:** 10.1186/s40658-016-0143-6

**Published:** 2016-04-22

**Authors:** Adam E. Hansen, Flemming L. Andersen, Sarah T. Henriksen, Alexandre Vignaud, Jan H. Ardenkjaer-Larsen, Liselotte Højgaard, Andreas Kjaer, Thomas L. Klausen

**Affiliations:** Department of Clinical Physiology, Nuclear Medicine and PET, Rigshospitalet, University of Copenhagen, Copenhagen, Denmark; Department of Electrical Engineering, Technical University of Denmark, Lyngby, Denmark; CEA, DRF, I2BM, NeuroSpin, UNIRS, CEA Saclay, Gif Sur Yvette, France

**Keywords:** PET/MRI, ^13^C magnetic resonance spectroscopic imaging, Hyperpolarization, Quantification, Interference

## Abstract

**Background:**

Integrated PET/MRI with hyperpolarized ^13^C magnetic resonance spectroscopic imaging (^13^C-MRSI) offers simultaneous, dual-modality metabolic imaging. A prerequisite for the use of simultaneous imaging is the absence of interference between the two modalities. This has been documented for a clinical whole-body system using simultaneous ^1^H-MRI and PET but never for ^13^C-MRSI and PET. Here, the feasibility of simultaneous PET and ^13^C-MRSI as well as hyperpolarized ^13^C-MRSI in an integrated whole-body PET/MRI hybrid scanner is evaluated using phantom experiments.

**Methods:**

Combined PET and ^13^C-MRSI phantoms including a NEMA [^18^F]-FDG phantom, ^13^C-acetate and ^13^C-urea sources, and hyperpolarized ^13^C-pyruvate were imaged repeatedly with PET and/or ^13^C-MRSI. Measurements evaluated for interference effects included PET activity values in the largest sphere and a background region; total number of PET trues; and ^13^C-MRSI signal-to-noise ratio (SNR) for urea and acetate phantoms. Differences between measurement conditions were evaluated using *t* tests.

**Results:**

PET and ^13^C-MRSI data acquisition could be performed simultaneously without any discernible artifacts. The average difference in PET activity between acquisitions with and without simultaneous ^13^C-MRSI was 0.83 (largest sphere) and −0.76 % (background). The average difference in net trues was −0.01 %. The average difference in ^13^C-MRSI SNR between acquisitions with and without simultaneous PET ranged from −2.28 to 1.21 % for all phantoms and measurement conditions. No differences were significant. The system was capable of ^13^C-MRSI of hyperpolarized ^13^C-pyruvate.

**Conclusions:**

Simultaneous PET and ^13^C-MRSI in an integrated whole-body PET/MRI hybrid scanner is feasible. Phantom experiments showed that possible interference effects introduced by acquiring data from the two modalities simultaneously are small and non-significant. Further experiments can now investigate the benefits of simultaneous PET and hyperpolarized ^13^C-MRI in vivo studies.

## Background

Integrated PET/MRI offers the combination of functional imaging of positron emission tomography (PET) and high soft-tissue contrast of anatomic magnetic resonance imaging (MRI) in a simultaneous acquisition. Clinical MRI is for the major part based on ^1^H nuclei, which are predominantly present in water. ^1^H-MRI has also functional capabilities, for instance to measure perfusion, characterize tissue cellularity through diffusion-weighted MRI, and study metabolites with magnetic resonance spectroscopy (MRS). The use of MRS imaging (MRSI) is, however, in general challenged by the low sensitivity of in vivo MRI.

Hyperpolarized MRI offers a signal increase by many orders of magnitude. Recently, the feasibility of using a ^13^C-labeled pyruvate substrate, hyperpolarized through the dynamic nuclear polarization method [1], for imaging of tumor metabolism in man was demonstrated [2]. Hence metabolic imaging based on ^13^C-MRSI has come within the reach of routine clinical use.

PET is an established clinical method for metabolic imaging. For example, in oncology, the tracer ^18^F-2-fluoro-2-deoxy-d-glucose (FDG) gives a measure of glucose metabolism. Since both ^13^C-MRSI and FDG-PET are metabolic imaging techniques, comparison studies are highly relevant [3] and have been pursued using sequential ^13^C-MRSI and PET [4, 5]. In the setting of integrated PET/MRI, simultaneous hyperpolarized ^13^C-MRSI and PET could become a reality. Such a setup could be very important for generation of new knowledge in physiology and pathophysiology as well as demonstrating potential clinical benefits of ^13^C-MRSI.

A prerequisite for the use of simultaneous PET and MRI is the absence of interference between the two systems for reviews; see, e.g., [6–8]. Absence of interference has been documented for a clinical whole-body system using simultaneous ^1^H-MRI and PET [9] but never for ^13^C-MRS(I) and PET. Radio frequency (RF) electromagnetic radiation, which is both transmitted and picked up by MRI coils, is a well-known potential source of interference both from MRI to PET and vice versa [7]. PET systems based on solid-state photodetectors have a major part of the electronics placed in close proximity to the MRI RF system. To avoid cross-talk, PET electronics is shielded by a conducting material [10–16]. The shielding material can induce eddy currents and magnetic field inhomogeneities, and the thickness of the material must therefore be minimized [11]; e.g., for a human brain PET insert, a 10-μm copper shield was used [15]. The magnetic resonance frequency is approximately four times lower for ^13^C than that for ^1^H due to the lower gyromagnetic ratio. Shielding efficiency depends on the square root of the frequency of the electromagnetic radiation and can be characterized by a penetration depth. Therefore, shielding optimized for ^1^H-MRI may not be similarly efficient for ^13^C-MRSI. Apart from shielding effects, also the magnetic field gradients employed for spatial encoding can potentially introduce noise in the PET measurement system [7]. Due to the lower gyromagnetic ratio, the amplitude of the imaging gradients may be increased. The lower ^13^C resonance frequency and as well the corresponding need for higher imaging gradients imply that performance measurements for simultaneous ^1^H-MR(S)I and PET cannot be directly translated to simultaneous ^13^C-MRSI and PET.

Thus, the aim of the present work is to demonstrate the feasibility of simultaneous PET and ^13^C-MRSI in an integrated whole-body PET/MRI hybrid scanner. Phantom experiments were carried out to investigate if simultaneous ^13^C-MRSI and PET acquisition is possible without influencing the PET and ^13^C-MRSI quantification. Furthermore, we aimed to demonstrate that the system is capable of hyperpolarized ^13^C-MRSI.

## Methods

### Experimental setup

Combined PET and MRI were performed using an integrated PET/MRI system (Siemens Biograph mMR, Erlangen, Germany).

**Fig. 1:**
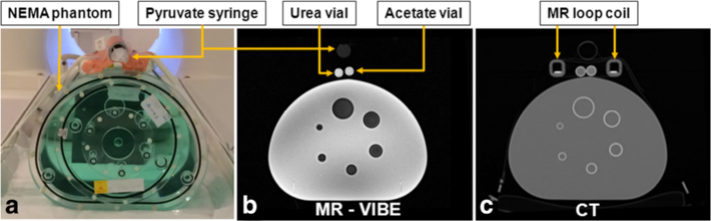
NEMA phantom setup. **a** Photo of setup with PET NEMA phantom and pyruvate syringe for hyperpolarized ^13^C-MRSI marked with arrows. **b** ^1^H-MRI with urea and acetate vials for thermally polarized ^13^C-MRSI marked with arrows. **c** CT with cross section of ^13^C loop coil marked with arrows

**Fig. 2:**
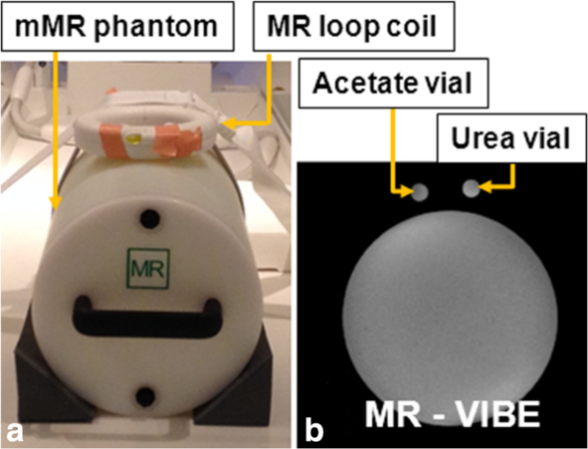
mMR water phantom setup. **a** Photo of setup with mMR water phantom. The ^13^C loop coil is marked with arrows. **b** ^1^H-MRI with urea and acetate vials marked with arrows

The phantom setups employed are shown in Figs. 1 and 2. An overview of all measurements is shown in Table 1. The PET performance (Fig. 1) was evaluated using a body-mimicking phantom (PTW, Freiburg, Germany) which is part of image quality tests according to the standard of the National Electrical Manufacturers Association (NEMA). The background part of the NEMA phantom was filled with demineralized water with added NaCl (5 g/L) and NiSO_4_ (3.75 g/L) for improving MR image quality compared to pure water [8]. A set of hollow spheres was filled with an aqueous solution of [^18^F]-FDG (40.11 kBq/mL). The background had an activity of 5.18 kBq/mL. Activities were measured by a well counter.

**Table 1:** Overview of measurements performed

Scan number	^13^C-MRSI acquisition	PET acquisition	PET gantry	FDG activity	Phantom	Comparisons
1, 3, 5, 7, 9, 11, 13, 15	ON	ON	ON	34–16 MBq	NEMA	PET, ^13^C-MRSI acq. ON versus OFF
2, 4, 6, 8, 10, 12, 14, 16	OFF	ON	ON			
17–20, 25–28	ON	OFF	ON	41–31 MBq	mMR water	^13^C-MRSI, PET gantry ON versus OFF; with high activity
21–24	ON	OFF	OFF			
29–32, 37–40	ON	OFF	ON	0.21–0.18 MBq		^13^C-MRSI, PET gantry ON versus OFF; with low activity
33–36	ON	OFF	OFF			

Scan numbers are consecutive in time. The total FDG activity of the phantom is given at the start and end of each measurement series (1–16, 17–28, and 29–40, respectively)

^1^H-MRI utilized the built-in RF body coil. ^13^C-MRI utilized a transmit/receive 12-cm loop coil (RAPID Biomedical, Würzburg, Germany) placed on top of the NEMA phantom. Inside the loop coil were placed two 5.5-mL vials of 4.0 M [^13^C]urea and 4.0 M [1-^13^C]acetate doped with gadolinium (Dotarem^®^) (0.23 %, *v*/*v*). The vials were centered vertically above the set of hollow spheres. A cardboard holder above the loop coil was used to place a syringe with hyperpolarized [1-^13^C]pyruvate.

For later alignment with CT data, a total of six fish oil capsules were placed at various positions at the phantom.

The ^13^C-MRSI performance was mainly evaluated using the phantom setup shown in Fig. 2. A cylindrical phantom supplied as a part of the PET/MRI system (mMR water, 9.5 L volume) was filled with demineralized water added an aqueous solution of [^18^F]-FDG (16.2 kBq/mL). The 12-cm loop coil was placed on top of the mMR water phantom, with the urea and acetate vials inside the loop.

### PET/MR data acquisition, NEMA phantom

The NEMA phantom (Fig. 1) was placed on the patient table with the set of hollow spheres in the isocenter of the scanner. Only the hardware described above was present.

Following localizer scans, first, a calibration of the ^13^C MR flip angle was performed using the urea vial. A series of free induction decay measurements with increasing transmitter voltage was acquired (repetition time (TR) 10,000 ms, echo time (TE) 0.35 ms, (nominal) flip angle 90°, pulse duration 0.50 ms, bandwidth 3000 Hz, transmitter voltage 10 to 90 V in steps of 10 V). Chemical shift spectra were centered on the [^13^C]urea peak. A sinusoidal fit of the [^13^C]urea peak height versus transmitter voltage was used to determine the relation between transmitter voltage and flip angle.

To evaluate the effect of simultaneous ^13^C-MRSI on the PET, a total of 16 PET list mode acquisitions were performed (1 bed position). During PET acquisitions with scan numbers 1, 3, 5, 7, 9, 11, 13, and 15, simultaneous ^13^C-MRSI was carried out; the remaining PET acquisitions were MR silent. The PET acquisition time was 6 min for scan numbers 1–8 and 8 min for scan numbers 9–16, to (partially) compensate for the decay of the tracer. During the first 58 s of each PET acquisition, vendor-supplied Dixon-VIBE MRI intended for MR attenuation correction (MRAC) was performed.

For ^13^C-MRSI, a chemical shift imaging (CSI) sequence was used (TR 130 ms, TE 2.3 ms, flip angle 29°, bandwidth 4000 Hz, 1024 points in spectrum, field-of-view 200 mm, voxel size 12.5 × 12.5 × 23.0 mm^3^). The sequence used a 16 × 16 grid and elliptical k-space sampling. Chemical shift spectra were centered on the [^13^C]urea peak. The CSI sampling time was 19 s and was repeated for the remainder of the PET acquisition following the Dixon MRI.

After the ^13^C-MRSI and PET acquisitions, a ^1^H-MRI VIBE sequence (TR 4.18 ms, TE 1.6 ms, flip angle 5.0°, bandwidth 540 Hz/pixel, field-of-view 350 mm, voxel size 1.6 × 1.6 × 3.0 mm^3^) was obtained as a structural reference.

### Hyperpolarization experiment

At the end of the PET/MRI NEMA phantom scan session, a hyperpolarization experiment was carried out. Hyperpolarized ^13^C-pyruvate was produced using the SpinLab system (GE Healthcare, Milwaukee, WI, USA). The sample consisted of [1-^13^C]pyruvic acid mixed with an electron paramagnetic agent (sample concentration 15 mM) manufactured by Syncom (Groningen, Netherlands, PN AH111501) under contract from General Electric. The dissolution media applied contained 0.1 g/L ethylenediaminetetraacetic acid disodium salt dehydrate (EDTA disodium salt, Sigma PN E4994) in water, and the neutralization media used to neutralize the hyperpolarized [1-^13^C]pyruvic acid sample prior to injection contained 0.72 M NaOH, 0.4 M Tris, and 0.1 g/L EDTA disodium salt in water. A 60-mL syringe with hyperpolarized ^13^C-pyruvate was placed in the abovementioned holder. Imaging took place immediately hereafter using the above CSI sequence with the flip angle reduced to 1°.

### CT data acquisition

Following the PET/MR NEMA phantom scan session, a CT (Siemens Biograph mCT) scan of the NEMA phantom including the coil was performed. The phantom was centered as in the PET/MR. The low-dose CT (120 kVp, 3-mm slices) scan was used for CT-based attenuation correction (CTAC).

### PET reconstruction

PET images of the NEMA phantom from both the PET and MR were reconstructed using vendor-supplied 3D OP-OSEM with 4 iterations, 21 subsets, and a 4-mm Gaussian filter. Voxel size was 2.08 × 2.08 × 2.03 mm^3^ with matrix 344.

For PET/MR CTAC, the low-dose CT image was used. First, the PET/CT patient table was manually removed using OsiriX (v. 4.1.2, Pixmeo, Geneva, Switzerland). CT images were subsequently aligned with the MR VIBE images using Minc (minctracc, McConnell Imaging Center, Montreal), whereupon the Hounsfield units were converted to linear attenuation coefficients using the bi-linear scaling of Carney et al. [17] as implemented in our PET/CT systems. The patient bed of the PET/MR is automatically included in the AC map by the vendor-supplied algorithm. No additional hardware elements were present during the PET/MR scan session. The first minute of the PET acquisition (during which simultaneous Dixon MRI was performed) was excluded from all reconstructions to study the possible effect of ^13^C-MRSI only.

### PET/MR data acquisition, mMR water phantom

The mMR phantom setup (Fig. 2) was placed on the patient table with the ^13^C loop coil in the isocenter of the scanner. A calibration of the ^13^C MR flip angle was performed using the urea vial with the same procedure as above.

To evaluate the effect of the PET data acquisition system on the ^13^C-MRSI, ^13^C-MRSI was performed with the PET gantry in an “ON” or “OFF” state. With the PET gantry in the OFF state, all PET electronics inside the Faraday cage of the PET/MRI examination room are switched off. This procedure can be accessed through the service module of the system. A total of 24 ^13^C-MRSI acquisitions were performed. During acquisitions with scan numbers 17–20 and 25–28, the PET gantry was ON; during acquisitions with scan numbers 21–24, the PET gantry was OFF. The ON-OFF-ON design was chosen as a slight temporal drift of the ^13^C-MRSI urea peak height was noted during preparation of the measurements. The ON-OFF-ON set of measurements was repeated 14 h later when the activity of the phantom had decayed to less than 1 % of its initial value. Please refer to Table 1 for an overview of measurements.

For ^13^C-MRSI, the above CSI sequence was used again (one repetition only). A ^1^H-MRI VIBE sequence with parameters as above was obtained as a structural reference.

### Evaluation of PET quantification

We report the average PET activity value (kBq/mL) of a spherical VOI with a diameter of 20 mm, centrally placed in the largest sphere (with an inside diameter of 37 mm). Background activity was measured using a spherical VOI with a diameter of 60 mm, placed in the center of the phantom. PET activity was decay corrected to the time of the filling of the phantom. In addition, the total number of trues for each PET acquisition on the PET/MR system was obtained from the list mode files (total prompts subtracted total randoms) and corrected for decay and the differences in acquisition times.

Percent differences between PET acquisitions obtained with ^13^C-MRSI active (ON) or not (OFF) were calculated using the ^13^C-MRSI OFF condition as the reference. Differences were evaluated using a two-sample *t* test.

### Evaluation of ^13^C-MRSI quantification

Peak heights of [1-^13^C]acetate, [^13^C]urea, and [1-^13^C]pyruvate (hyperpolarized experiment only) were quantified for every MRSI voxel using a general linear model implemented in MATLAB (MathWorks, Natick, MA, USA). The estimation was performed in the time domain. Metabolite maps were interpolated on a 128 × 128 grid for visual presentation.

The ^13^C-MRSI noise was derived from the background part of the ^13^C spectrum (from −2000 to −1000 Hz and from 1000 to 2000 Hz) in voxel (1,1) at the corner of the ^13^C-MRSI grid, far from the ^13^C phantoms, which was verified to have no signal. The noise was estimated as the standard deviation of the real part of the signal, multiplied by the square root of the number of repetitions of the MRSI. ^13^C-MRSI signal-to-noise ratio (SNR) was estimated for urea and acetate using the voxel with maximum signal. The signal was defined as the integrated peak area [18] in an interval of ±250 Hz around the peak.

Percent differences of SNR between ^13^C-MRSI acquisitions for the mMR water phantom obtained with PET gantry “ON” or “OFF” were calculated using the OFF condition as the reference. Differences were evaluated using a two-sample *t* test. Differences were evaluated both with and without high activity in the gantry (see Table 1).

### Statistical evaluation

Data are reported with 95 % confidence intervals (CI), and a *p* value of 0.05 was considered significant.

## Results

On a general note, PET and ^13^C-MRSI data acquisition could be performed simultaneously without any practical issues.

### PET quality

**Fig. 3:**
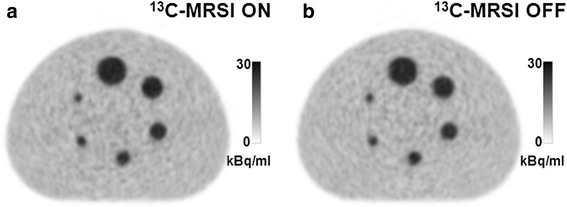
Examples of PET during ^13^C-MRSI ON and OFF. PET with **a** simultaneous ^13^C-MRSI (scan number 1) and **b** without any MRI performed (scan number 2)

**Fig. 4:**
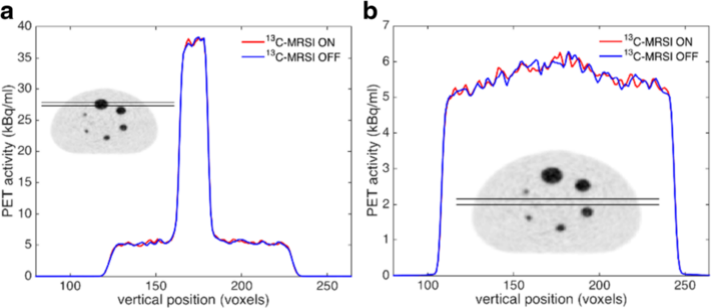
PET profiles during ^13^C-MRSI ON and OFF. PET line plots of voxels between the *black lines of the inserts*, through **a** the largest sphere (single slice) and **b** background region (10 slices). Conditions of simultaneous ^13^C-MRSI ON and OFF are shown as *red* and *blue* colors, respectively

**Fig. 5:**
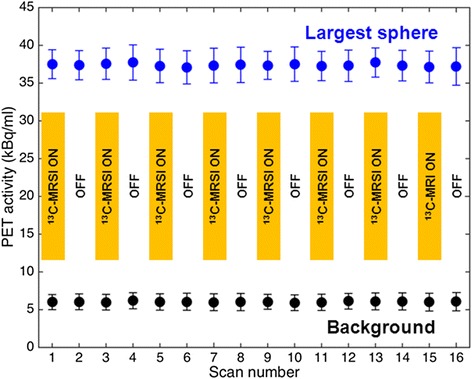
PET activity for all NEMA phantom measurements. Measured PET activity in the largest sphere (*blue points*) and in a background region of the phantom (*black points*). *Error bars* are standard deviations on estimates. For odd PET scan numbers, simultaneous ^13^C-MRSI was performed, as indicated by the *yellow bars*

As an example of PET quality, reconstructed transverse images from scan numbers 1 (^13^C-MRSI “ON”) and 2 (^13^C-MRSI “OFF”) are shown in Fig. 3. Line plots of average activity during ^13^C-MRSI ON and OFF conditions are shown in Fig. 4. No artifacts from the simultaneous ^13^C-MRSI acquisition were discerned. However, both background profiles show a peak towards the center. Figure 5 shows PET activity in the largest sphere and the background for all 16 PET acquisitions. There was no visible time dependence or dependence on whether the ^13^C-MRSI was ON or OFF.

The results are summarized in Table 2. The average difference in PET activity between acquisitions with ^13^C-MRSI ON and OFF was 0.83 (largest sphere) and −0.76 % (background). The differences were not significant (*p* = 0.18, CI (−0.42 to 2.07 %) and *p* = 0.16, CI (−1.85 to 0.33 %)) for the largest sphere and background, respectively. The average difference in net trues was −0.01 % and was not significant (*p* = 0.97, CI (−0.38 to 0.37 %)).

**Table 2:** Summary of PET measurements

	^13^C-MRSI ON (*n* = 8)	^13^C-MRSI OFF (*n* = 8)
PET activity, largest sphere	37.62 ± 0.49 kBq/mL	37.31 ± 0.38 kBq/mL
PET activity, background	6.02 ± 0.04 kBq/mL	6.06 ± 0.08 kBq/mL
Net trues	208,943 ± 723	208,959 ± 747

Measurements are performed on PET/MR with and without simultaneous ^13^C-MRSI

### ^13^C-MRSI quality

**Fig. 6:**
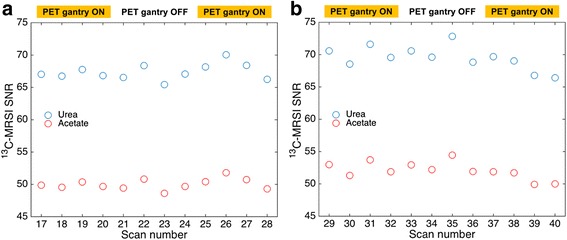
^13^C-MRSI SNR for mMR water phantom measurements. Plots show urea (blue *symbols*) and acetate (*red symbols*) SNR for ^13^C-MRSI scan numbers 17 to 40. For scan numbers 21–24 and 33–36, the PET gantry was switched off, as indicated by the *yellow bar*. The PET activity was high and low for scan numbers 17–28 (**a**) and scan numbers 29–40 (**b**), respectively

Figure 6 shows [^13^C]urea and [1-^13^C]acetate SNR for ^13^C-MRSI acquisitions using the mMR water phantom. There was no apparent dependence on whether the PET gantry was OFF or ON, neither in the condition with PET activity high (Fig. 6a) nor low (Fig. 6b). However, a tendency to an overall temporal drift can be noticed in Fig. 6b. The average difference in SNR between acquisitions with high PET activity and PET gantry ON versus OFF was 1.21 % and was not significant (*p* = 0.31, CI (−1.27 to 3.67 %)) for [^13^C]urea and was 1.15 % and not significant (*p* = 0.29, CI (−1.13 to 3.42 %)) for [1-^13^C]acetate. The average difference in SNR between acquisitions with low PET activity and PET gantry ON versus OFF was −2.03 % and was not significant (*p* = 0.21, CI (−5.51 to 1.38 %)) for [1-^13^C]urea and was −2.28 % and not significant (*p* = 0.15, CI (−5.61 to 0.98 %)) for [1-^13^C]acetate. Further examination of the data revealed that neither noise level nor integrated peak areas were significantly different between PET gantry OFF and ON conditions.

**Fig. 7:**
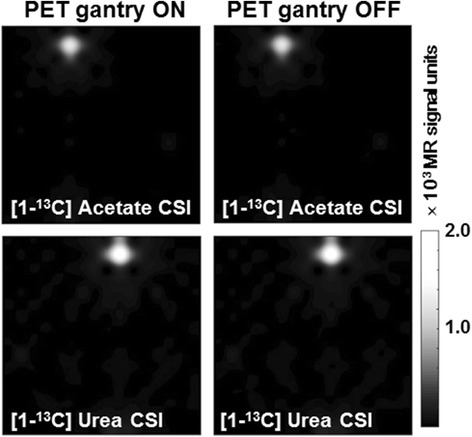
Examples of ^13^C-MRSI during PET gantry ON and OFF. ^13^C-MRSI obtained with the PET gantry OFF (*left column*) and with the PET gantry ON (*right column*). Top row is acetate, bottom row urea metabolite images

**Fig. 8:**
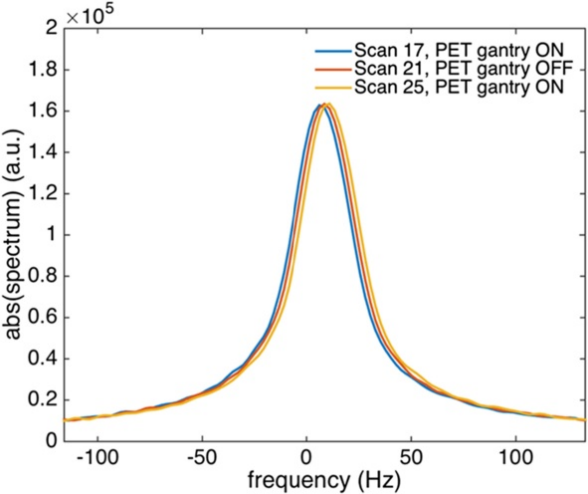
Examples of ^13^C spectroscopy during PET gantry ON and OFF. Plots show ^13^C spectra under conditions of the PET gantry ON and OFF, as denoted in the legend. Spectra are from the voxel with the maximum urea signal and zoomed on the urea peak to highlight the small differences of the spectra

In Fig. 7, we show examples of the ^13^C-MRSI obtained with the mMR water phantom with (scan number 17) and without (scan number 21) the PET gantry switched on. No artifacts from the PET electronics were discerned. Figure 8 shows examples of ^13^C spectra zoomed on the [^13^C]urea peak. The only visible difference between PET gantry ON and OFF conditions is a small drift in peak position.

**Fig. 9:**
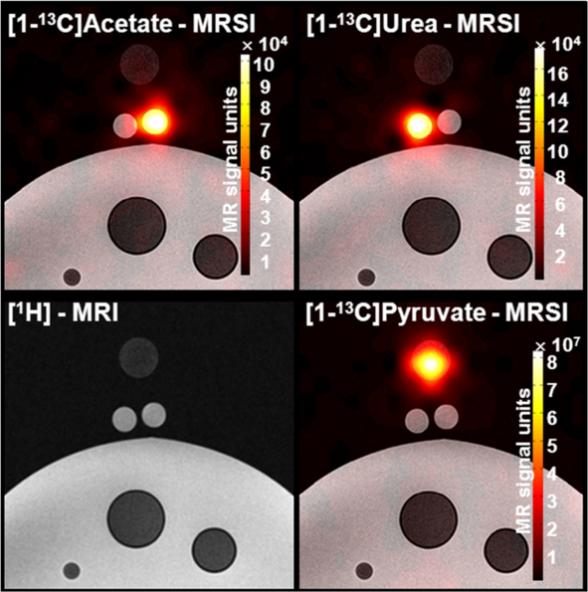
Examples of ^13^C-MRSI. ^13^C-MRSI metabolite images (*color*, *scale bar inserted*) overlaid on ^1^H-MRI (*lower left*). *upper row*: Acetate and urea metabolite images. *lower right*: Image of hyperpolarized pyruvate

Finally, in Fig. 9 (lower right), we show an example of hyperpolarized ^13^C-MRSI with [1-^13^C]pyruvate.

## Discussion

The presented phantom data demonstrates that simultaneous PET and ^13^C-MRSI in a whole-body PET/MRI scanner is possible. Interference effects introduced by acquiring data from the two modalities simultaneously are small and non-significant. Hence, full advantage can be taken of the integrated PET/MRI scanner design. Furthermore, the integrated PET/MRI system was proven capable of hyperpolarized ^13^C-MRSI.

A simultaneous acquisition of PET and ^13^C-MRSI will reduce total examination time and minimize subject motion between the imaging modalities, which is likely to be beneficial for in vivo studies. Also, functional imaging can be performed without a delay between the modalities, thus ensuring that the physiologic state of the subject is unchanged. This is particularly desirable when ^13^C-MRSI is combined with PET tracers using a short uptake time and in cases where a pharmacological challenge or another intervention is employed.

The PET results presented here using a standard quality assurance phantom shows no effect of simultaneous ^13^C-MRSI (Figs. 3 and 4). The statistical analysis of our repeated measurements (Fig. 5) allows us to conclude that the maximum possible quantitative PET estimation error due to interference from the ^13^C-MRSI is below 2 % (95 % CI). That can be considered a sufficiently low error for clinical and research purposes.

The CSI sequence used to test interference with PET is a typical type of sequence for hyperpolarized MRSI. The RF flip angle was set to 29°, corresponding to the Ernst angle for a typical tissue longitudinal MR relaxation time (*T*_1_) of 1000 ms, giving a maximum signal for a thermal polarization. In case of hyperpolarized MRI, most often a lower flip angle is chosen (e.g., 10° in Ref. [2]) to preserve the hyperpolarization as long as possible. Hence in practice, possible interference effects caused by RF radiation will be smaller than in the present experiment.

When MRI is not performed, no RF radiation is transmitted and the magnetic field is constant. Conversely, there is no fundamental change of state of the PET detector system between situations where imaging data is collected or not. A possible RF interference from the PET detector system to the ^13^C-MRSI would depend on the PET gantry being powered on or off. However, no effect of power state is observed (Figs. 6, 7, and 8). The maximum ^13^C-MRSI SNR error due to PET gantry power state is below −6 to +4 % (95 % CI) for all measurements. A tendency to temporal drift in the measurements, seemingly unrelated to the power state (see Fig. 6b), could have inflated the error estimation. The ON-OFF-ON design of the experiment will compensate for a linear trend of system instability only. Overall though, a stability of a few percent on ^13^C-MRSI SNR in a single 19-s acquisition as used here can be considered satisfactory.

The present study was not designed to investigate a hypothetical overall (i.e., PET acquisition and activity independent) degradation of the ^13^C-MRSI due to the PET system. For example, the presence of the PET detector system could degrade the static magnetic field inhomogeneity; however, this was shown not to be the case for the PET/MRI system used here by Delso et al. [9]. The focus of the present work was on RF effects which depend on the gyromagnetic ratio and hence potentially differ from ^1^H- to ^13^C-based imaging.

Importantly, the ^13^C-MRSI quality in this integrated PET/MRI setup was sufficient to perform metabolic imaging using hyperpolarization in a phantom setting (Fig. 9) and also recently for in vivo experiments [19, 20].

We noticed an unexpected non-uniform activity in the background region of the NEMA phantom (Fig. 4b). The non-uniformity could originate from suboptimal attenuation or scatter correction of the setup and deserves further investigation. However, the profile appeared to be independent of the ^13^C-MRSI acquisition. Further challenges specific to simultaneous PET and ^13^C-MRSI include characterization and correction for photon attenuation due to ^13^C coils. Preliminary investigations at our institution indicate that this effect can change PET activity values with up to 10 %, however, without any change in visual interpretation of images.

## Conclusions

The present work demonstrated the feasibility of simultaneous PET and ^13^C-MRSI in an integrated whole-body PET/MRI hybrid scanner. Phantom experiments showed that simultaneous ^13^C-MRSI and FDG-PET acquisition is possible without influencing the PET quantification. Furthermore, the system is capable of hyperpolarized ^13^C-MRSI. Hereby, the way is paved for simultaneous PET and hyperpolarized ^13^C-MRI in vivo studies.
